# Caregiver-reported experience of integrated care for children with special health care needs and its sociodemographic, health- and network-related correlates in Germany: Cross-sectional results from the PART-CHILD cohort

**DOI:** 10.1186/s12913-026-15276-6

**Published:** 2026-08-08

**Authors:** Kerstin Bohnert, Angélique Herrler, Freia De Bock, Vera Araújo-Soares, Michael Eichinger

**Affiliations:** 1 Division of Public Health, Social and Preventive Medicine, Center for Preventive Medicine and Digital Health, Medical Faculty Mannheim, Heidelberg University, Mannheim, Germany; 2 Department of General Pediatrics, Neonatology and Pediatric Cardiology, Medical Faculty and University Hospital Düsseldorf, Heinrich Heine University Düsseldorf, Düsseldorf, Germany; 3 Institute of Medical Sociology and Rehabilitation Science, Charité - University Medicine Berlin, Corporate member of Freie Universität Berlin and Humboldt-Universität zu Berlin, Berlin, Germany; 4 Division for Prevention of Cardiovascular and Metabolic Diseases, Center for Preventive Medicine and Digital Health, Medical Faculty Mannheim, Heidelberg University, Mannheim, Germany; 5 Institute of Medical Biostatistics, Epidemiology and Informatics, University Medical Center of the Johannes Gutenberg University Mainz, Mainz, Germany

**Keywords:** Care coordination, Care networks, Caregivers, Family-centered care, Integrated care, Special health care needs, Team communication, Unmet needs

## Abstract

**Background:**

Children with special health care needs (CSHCN) often rely on cross-sectoral care networks involving medical, therapeutic, educational, and social services. However, key dimensions of integrated care, including cross-sectoral communication and attention to family impact, remain challenging. Understanding factors that shape caregiver-reported experience of integrated care can inform program development to strengthen integrated care. We aimed (1) to quantify caregiver-reported experience of integrated care and (2) to examine associations between dimensions of caregiver-reported experience of integrated care and sociodemographic, health- and network-related correlates.

**Methods:**

We analyzed cross-sectional data from the nationwide PART-CHILD cohort. Caregivers of CSHCN with predominantly neurological, developmental, and behavioral conditions were recruited in specialized outpatient facilities in Germany and completed questionnaires. Caregiver-reported experience of integrated care was assessed using the German Pediatric Integrated Care Survey, yielding composite scores for *Team quality and communication* and *Attention to family impact*. Associations with sociodemographic characteristics, reason for seeking care, unmet needs, and care network size were examined using multivariable linear regression models.

**Results:**

Caregivers of CSHCN (*n* = 459) reported moderate *Team quality and communication* (mean 4.1 ± 1.1) and low *Attention to family impact* (2.3 ± 1.1). Higher unmet needs were associated with lower *Team quality and communication* (1−4 unmet needs: β = −0.28, 95% CI: −0.52 to − 0.04; ≥5 unmet needs: β = −0.83, 95% CI: −1.28 to − 0.38; reference: none). Cognitive impairment (β = 0.36, 95% CI: 0.03 to 0.69; reference: physical impairment) and larger care networks (4 − 5 providers: β = 0.33, 95% CI: 0.08 to 0.58; 6 − 10 providers: β = 0.38, 95% CI: 0.07 to 0.70; reference: 2–3 providers) were associated with higher *Attention to family impact*.

**Conclusion:**

Strengthening integrated care for CSHCN may require greater attention to family-level concerns, which were rated low in this sample. The association between unmet needs and *Team quality and communication* suggests a close link between perceived integration and needs-based service delivery. Correlates of *Attention to family impact*, including impairment type and care network size, warrant further investigation and may help identify target groups for interventions to strengthen family-centered services.

**Trial registration:**

The study was prospectively registered in the German Clinical Trials Register on 16 November 2018 (ID: DRKS00015054).

**Supplementary Information:**

The online version contains supplementary material available at 10.1186/s12913-026-15276-6.

## Background

Children and youth (hereinafter referred to as *children*) with special health care needs (CSHCN) represent a heterogeneous population with chronic conditions, requiring care beyond that usually needed by children [1, 2]. Their care networks typically consist of multiple providers from different sectors, including medical, therapeutic, educational, and social services [3, 4]. For many families, navigating these complex networks is challenging, requires management skills, and can lead to parental exhaustion, delayed care, and unmet service needs. These challenges are particularly pronounced for families affected by social disadvantage [5] and for children with neurological, developmental, and behavioral conditions [6], whose complex care needs and limited ability to compensate for fragmented cross-sectoral care may further increase the burden on families.

Integrated care is a key approach to overcome fragmented service provision by improving coordination, communication, and continuity of care between providers and sectors, thus improving care delivery and patient outcomes [7, 8]. Although models of integrated care for CSHCN have been established in several health care systems and policy attention has increased, implementation of integrated care remains heterogeneous, and care networks in many countries continue to lack effective, well-coordinated services [9, 10].

Understanding which aspects of integrated care matter most to families requires consideration of parents’ (or more broadly *caregivers’*) perspectives. Previous research has identified cross-sectoral communication and collaboration, as well as attention to family needs, as key dimensions of effective integrated care, which are closely linked to caregivers’ experiences of care quality [11–14]. Hereafter, we refer to these dimensions collectively as *experience of integrated care* (EIC) to avoid redundancy. Qualitative studies have provided in-depth insights into family perspectives on service integration [15], while population-based surveys have examined disparities in access to and unmet needs for care coordination by ethnicity [16], socioeconomic and insurance status [17] and variation in communication between health care providers and the educational sector, including schools, by child health condition [18].

The present study addresses several important knowledge gaps. First, whereas most previous studies have assessed caregiver-reported EIC using single items or ad hoc composite measures, we used a validated, multidimensional instrument to capture EIC as a multifaceted construct [19]. This approach enables the identification of specific dimensions of integrated care that remain insufficiently addressed in cross-sectoral care networks. Second, existing evidence is largely derived from the United States, with limited evidence from other health care systems [20, 21]. By examining EIC in Germany, the present study extends the international evidence base to a European health care system based on statutory health insurance. Finally, the study comprehensively characterizes families experiencing particularly low levels of integrated care, thereby helping to identify target groups for interventions to strengthen coordinated, family-centered care.

In this study, we aimed (1) to quantify caregiver-reported EIC within care networks of CSHCN with predominantly neurological, developmental, and behavioral conditions in Germany, focusing on the dimensions *Team quality and communication* and *Attention to family impact*, and (2) to examine associations between these dimensions of caregiver-reported EIC and sociodemographic, health- and network-related potential correlates.

## Methods

### Study design and setting

In this study, we used data from the PART-CHILD cohort, collected between February 2019 and October 2020 [22]. The cohort was part of a stepped-wedge cluster randomized trial evaluating an intervention to improve participation-centered care for CSHCN in Social Pediatric Centers (*Sozialpädiatrische Zentren*, henceforth referred to as *SPC*). Fourteen of the 156 SPC in Germany, distributed across eight of the country’s 16 federal states, participated in the first follow-up survey. The participating SPC reflected the structural heterogeneity of SPC in Germany with respect to size, additional financial resources and staff work experience [23]. SPC are statutorily mandated facilities providing secondary and tertiary outpatient care for children with complex chronic conditions. Led by pediatricians, they offer diagnostic services and treatment for CSHCN with predominantly neurological, developmental, and behavioral conditions as well as disabilities, while also supporting their caregivers [24, 25]. As there was no evidence of an intervention effect on primary or secondary outcomes, we treated the data as observational (unpublished data).

The study was approved by the Medical Ethics Review Board of the Medical Faculty Mannheim at Heidelberg University (2018–529N-MA) and the local ethics committees of all participating SPC. Written informed consent to participate in the study was obtained from all caregivers. The study was prospectively registered in the German Clinical Trials Register on 16 November 2018 (ID: DRKS00015054). Reporting of the study is based on the STROBE reporting guideline [26].

### Participants and data collection

Caregivers of CSHCN with predominantly neurological, developmental, and behavioral conditions receiving care at one of the participating SPC were invited to participate by health care providers at the SPC. Instead of using a standardized CSHCN screener, eligibility was based on admission to an SPC, serving as a pragmatic proxy for CSHCN status. SPC provide interdisciplinary outpatient care for children with chronic, complex, and often multimorbid conditions that exceed the scope of routine pediatric care, and thereby predominantly serve children who meet the conceptual definition of CSHCN [1, 2]. Caregivers were eligible to participate if they (1) were able to complete questionnaires in German, (2) had a child aged between 0 and 16 years, and (3) were expected to have at least four appointments at one of the SPC during the upcoming two years. Caregivers completed paper-and-pencil or online questionnaires. Respondent type was categorized as mother, father, or other caregiver. Information on the child’s living arrangement (e.g., biological family, kinship care, foster care, or residential care) was not collected.

### Collected data

In this section, we provide a concise summary of the collected data. A detailed overview of all variables and their operationalization is provided in Additional file 1.

#### Caregiver-reported experience of integrated care

Caregiver-reported EIC was assessed using the Pediatric Integrated Care Survey in German (PICS-D), a self-report instrument. In the psychometric evaluation, the PICS-D showed good structural validity and internal consistency [19]. It comprises 11 validated items grouped into two composite scores:

1. The **Team quality and communication** score focuses on the perceived quality of collaboration and communication within care networks, including how members of care networks interact with caregivers and involve them in care- and health-related decisions (e.g., ‘In the past 12 months, how often did your child’s care team members explain things in a way that you could understand?’).
2. The **Attention to family impact** score focuses on the perceived extent to which members of the care network consider how care decisions affect the family (e.g., ‘In the past 12 months, how often have your child’s care team members talked with you about how health care decisions for your child will affect your whole family?’).

All items were rated on a six-point Likert scale ranging from 1 (never) to 6 (always), with higher scores indicating better EIC. The 12-month reference period follows the original PICS [19, 27].

#### Sociodemographic, health- and network-related correlates of caregivers and CSHCN

Sociodemographic correlates included sex, age, German as first language and highest level of education of caregivers as well as sex and age of CSHCN. Due to data protection regulations, age could only be assessed as age groups. Educational attainment of both caregivers was assessed using the International Standard Classification of Education (ISCED), combining general and vocational training, and classified as basic (ISCED 0–2: no formal schooling up to lower secondary education, e.g., compulsory schooling), intermediate (ISCED 3–4: upper secondary and post-secondary non-tertiary education, e.g., apprenticeship, secondary school certificate) and high (ISCED 5–8: short-cycle tertiary education and above, e.g., university degree) [28]. We analyzed education levels of caregivers separately, given evidence that each caregiver’s education may show distinct associations with child health [29] and maternal education may be particularly relevant given that mothers are typically the primary caregivers of CSHCN [30]. The reason for seeking care was recorded as free-text and categorized. Care network size was assessed as the total number of service providers involved in the child’s care in the last 12 months. Unmet needs were investigated using the Child Health Care–Satisfaction, Utilization and Needs (CHC-SUN) scale [31] for non-physician services such as physiotherapy, self-help groups, or medical aids. The number of perceived unmet needs was categorized as none, 1–4, and ≥5. The highest category combined infrequent values to ensure a minimum cell size of 30 observations for stable regression estimates.

### Data analysis

All statistical analyses were conducted with R Studio (version 2026.07.1) using R (version 4.6.0; The R Foundation for Statistical Computing Platform). The analytic sample comprised caregivers reporting at least two health care providers in the child’s care network, as team communication and collaboration cannot be meaningfully assessed in networks involving only one provider. Caregivers in the study sample reporting only one health care provider were excluded on this basis.

#### Descriptive statistics

We investigated sample characteristics using means and standard deviations (SD) for continuous variables as well as absolute and relative frequencies for categorical variables. Based on guidance from the PICS-D validation study [19], the EIC composite scores for *Team quality and communication* and *Attention to family impact* were calculated as the mean of all items within each scale, allowing up to 20% missing values per scale.

#### Regression analyses

We fitted linear regression models to examine associations between sociodemographic, health- and network-related correlates and the outcomes *Team quality and communication* (Model 1) and *Attention to family impact* (Model 2). First, we used univariable models to assess each predictor separately. Second, we fitted multivariable models adjusted for all covariates.

Missing values were addressed using multiple imputation by chained equations (MICE) as the primary analytical approach, assuming a missing-at-random mechanism [32]. Missingness patterns are reported in Table 1 by comparing the study, analytic, and complete-case samples. Multiple imputation was performed using the mice package in R [33]. The number of imputations was set to m = 40, approximately corresponding to the proportion of incomplete cases in the analytic sample (33.1%) [34]. Predictive mean matching was used for continuous variables, and logistic or polytomous regression for binary and categorical variables, with 30 iterations per chain.

In addition to the regression covariates, the imputation model included auxiliary variables expected to be associated with the missingness mechanism or the regression covariates: (1) SPC identifier, (2) duration of care at the SPC (years since first appointment), (3) pandemic phase, (4) respondent type (mother, father, or other caregiver), (5) supplementary PICS-D items on caregiver-reported EIC (e.g., ‘How often did you have difficulties or delays getting medical or social services for your child because you had trouble getting the information you needed?’), and (6) caregiver-reported satisfaction with primary pediatric, SPC, and emergency outpatient care. Convergence was assessed visually using trace plots. Regression models were fitted to each imputed dataset, and parameter estimates were pooled according to Rubin’s rules [35]. A detailed overview of all variables included in the imputation model, including their classification as outcomes, regression covariates, or auxiliary variables, is provided in Additional file 2.

Model fit was assessed using R², pooled across imputations [36]. Regression coefficients (β) are reported with 95% confidence intervals (CI). Associations were considered statistically significant when the 95% CI excluded zero. Model assumptions were assessed using residual diagnostics from five randomly selected imputed datasets [37]. Linearity and homoscedasticity were evaluated using residual plots, normality using Q-Q plots, heteroscedasticity using the Breusch-Pagan test, and multicollinearity using variance inflation factors (VIF <2 indicating acceptable levels [38]). Influential observations were identified using Cook’s Distance, with values exceeding conventional thresholds considered indicative of influential observations [39].

#### Sensitivity analyses

To assess the robustness of our findings, we performed several sensitivity analyses. First, we repeated the analyses using complete cases only, restricting the sample to the 335 caregivers with no missing values for any regression variable. Detailed results are provided in Additional file 3. Second, we fitted two-level linear mixed-effects models with random intercepts to account for the hierarchical data structure, with caregivers nested within SPC. The intraclass correlation coefficient (ICC) was used to quantify the proportion of variance attributable to between-SPC differences [40]. Third, we fitted fixed-effects models for SPC to account for site-specific differences. Finally, we conducted stratified analyses by survey completion period, grouped according to the pandemic phases in Germany [41].

## Results

### Descriptive statistics

In total, 501 caregivers of CSHCN participated in the first follow-up survey of the PART-CHILD cohort and were included in the study sample. The analytic sample comprised 459 caregivers who reported at least two providers in the health care network, and 335 caregivers were included in the complete-case sample. Sample characteristics including caregiver-reported EIC are summarized in Table 1. The majority of respondents were mothers (86%), followed by fathers (7%), and other caregivers (7%). CSHCN in the sample had a broad spectrum of neurological, developmental, and behavioral conditions including epilepsy, cerebral palsy, autism spectrum disorder, and attention deficit hyperactivity disorder.

Caregivers of CSHCN reported moderate *Team quality and communication* (mean 4.1 ± 1.1) and low *Attention to family impact* (2.3 ± 1.1).

**Table 1:** Sample characteristics of caregivers and CSHCN

	Study sample (*n* = 501)	Analytic sample (*n *= 459)	Complete-case sample (*n* = 335)
**Team quality and communication** ^a^	4.1 (± 1.1)	4.1 (± 1.1)	4.2 (± 1.1)
Missing	59	34	-
**Attention to family impact** ^a^	2.3 (± 1.1)	2.3 (± 1.1)	2.3 (± 1.1)
Missing	48	27	-
**Potential correlates** ^b^			
Sex of caregiver			
Female	417 (92.3)	385 (92.1)	311 (92.8)
Male	35 (7.7)	33 (7.9)	24 (7.2)
Missing	49	41	-
Age of caregiver			
≤18 years	0 (0.0)	0 (0.0)	0 (0.0)
19–25 years	0 (0.0)	0 (0.0)	0 (0.0)
26–30 years	23 (5.0)	19 (4.5)	15 (4.5)
31–35 years	77 (16.8)	74 (17.5)	58 (17.3)
36–40 years	137 (29.9)	124 (29.2)	102 (30.4)
41–45 years	123 (26.9)	114 (26.9)	89 (26.6)
≥46 years	98 (21.4)	93 (21.9)	71 (21.2)
Missing	43	35	-
German as first language			
Yes	395 (86.8)	373 (88.6)	297 (88.7)
No	60 (13.2)	48 (11.4)	38 (11.3)
Missing	46	38	-
Mothers’ highest level of education^c^			
Basic	34 (7.5)	28 (6.7)	21 (6.3)
Intermediate	321 (70.7)	298 (71.1)	230 (68.6)
High	99 (21.8)	93 (22.2)	84 (25.1)
Missing	47	40	-
Fathers’ highest level of education^c^			
Basic	38 (9.1)	36 (9.2)	30 (8.9)
Intermediate	276 (66.0)	261 (66.9)	224 (66.9)
High	104 (24.9)	93 (23.9)	81 (24.2)
Missing	83	69	-
Sex of CSHCN			
Female	141 (30.9)	129 (30.6)	93 (27.8)
Male	315 (69.1)	292 (69.4)	242 (72.2)
Missing	45	38	-
Age of CSHCN			
1–3 years	6 (1.2)	5 (1.1)	4 (1.2)
4–5 years	99 (21.6)	94 (22.2)	81 (24.2)
6–7 years	117 (25.5)	108 (25.5)	84 (25.1)
8–11 years	151 (32.9)	136 (32.1)	112 (33.4)
≥12 years	86 (18.8)	81 (19.1)	54 (16.1)
Missing	42	35	-
Reason for seeking care			
Physical impairment	90 (18.3)	83 (18.2)	65 (19.4)
Cognitive impairment	157 (31.9)	141 (30.9)	107 (31.9)
Physical and cognitive impairment	205 (41.5)	197 (43.2)	142 (42.4)
Only diagnostic assessments and therapies	40 (8.1)	35 (7.7)	21 (6.3)
Missing	9	3	-
Number of perceived unmet needs			
None reported	266 (53.1)	235 (51.2)	156 (46.5)
1−4 unmet needs	203 (40.5)	192 (41.8)	149 (44.5)
≥5 unmet needs	32 (6.4)	32 (7.0)	30 (9.0)
Missing	0	0	-
Size of care network			
None reported / 1 provider^d^	42 (8.4)	-	-
2–3 providers	148 (29.5)	148 (32.2)	101 (30.1)
4–5 providers	222 (44.3)	222 (48.4)	167 (49.9)
6–10 providers	89 (17.8)	89 (19.4)	67 (20.0)
Missing	0	0	-

CSHCN, children with special health care needs; ISCED, International Standard Classification of Education; PICS-D, German Pediatric Integrated Care Survey; SD, standard deviation

The study sample comprised all caregivers who completed the first follow-up survey. The analytic sample included all caregivers with at least two providers in the care network. The complete-case sample comprised caregivers with complete data on all variables in the regression analyses and was used for sensitivity analyses

^a^ We report the mean and standard deviation (SD) for the PICS-D scores *Team quality and communication* and *Attention to family impact* (possible range: 1–6). A higher score indicates better experience of integrated care

^b^ No. (%)

^c^ We used the International Standard Classification of Education (ISCED) to report the highest level of education

^d^ Families reporting no care network or only one provider (*n* = 42) were excluded from regression analyses, as team communication and collaboration cannot be meaningfully assessed in networks involving only one provider

### Regression analyses

The multivariable linear regression model (Table 2, model 1b) explained approximately 6.4% of the variance in the *Team quality and communication* score. Higher numbers of unmet needs were associated with lower *Team quality and communication* scores (1–4 unmet needs: β=−0.28, 95% CI: −0.52 to − 0.04; ≥5 unmet needs: β=−0.83, 95% CI: −1.28 to − 0.38; reference: no unmet needs).

The multivariable linear regression model (Table 3, model 2b) explained approximately 10.3% of the variance in the *Attention to family impact* score. Cognitive impairment (β = 0.36, 95% CI: 0.03 to 0.69; reference: physical impairment) and larger size of care network (4 − 5 providers: β = 0.33, 95% CI: 0.08 to 0.58; 6 − 10 providers: β = 0.38, 95% CI: 0.07 to 0.70; reference: 2 − 3 providers) were associated with higher *Attention to family impact* scores.

Model diagnostics indicated no major violations of linearity, normality, or homoscedasticity in either model. VIF showed no evidence of multicollinearity. No highly influential observations were identified. Univariable associations were largely consistent with multivariable results (Models 1a and 2a in Table 2 and Table 3).

**Table 2:** Associations between caregiver-reported *Team quality and communication* and sociodemographic, health- and network-related potential correlates

		1a: Univariable linear regression models	1b: Multivariable linear regression model
		β (95% CI)	β (95% CI)
Sex of caregiver	Female (Ref.)		
	Male	−0.18 (−0.59 to 0.23)	−0.21 (− 0.63 to 0.21)
Age of caregiver	≤35 years (Ref.)		
	36–40 years	0.00 (− 0.34 to 0.34)	0.03 (− 0.31 to 0.38)
	41–45 years	0.01 (− 0.33 to 0.34)	−0.03 (− 0.38 to 0.32)
	≥46 years	−0.18 (− 0.54 to 0.17)	−0.27 (− 0.66 to 0.13)
German as first language	Yes (Ref.)		
	No	−0.07 (− 0.46 to 0.32)	−0.13 (− 0.53 to 0.27)
Mothers’ highest level of education	Basic and intermediate (Ref.)		
	High	−0.15 (− 0.43 to 0.14)	−0.08 (− 0.39 to 0.23)
Fathers’ highest level of education	Basic and intermediate (Ref.)		
	High	−0.22 (− 0.50 to 0.06)	−0.20 (− 0.52 to 0.11)
Sex of CSHCN	Female (Ref.)		
	Male	−0.07 (− 0.33 to 0.19)	−0.06 (− 0.33 to 0.20)
Age of CSHCN	1–5 years (Ref.)		
	6–11 years	0.03 (− 0.25 to 0.31)	−0.03 (− 0.33 to 0.27)
	≥12 years	0.06 (− 0.31 to 0.42)	0.13 (− 0.28 to 0.54)
Reason for seeking care	Physical impairment (Ref.)		
	Cognitive impairment	−0.01 (− 0.34 to 0.31)	−0.07 (− 0.42 to 0.28)
	Physical and cognitive impairment	−0.22 (− 0.53 to 0.09)	−0.24 (− 0.56 to 0.08)
	Only diagnostic assessments and therapies	−0.06 (− 0.57 to 0.46)	−0.22 (− 0.75 to 0.32)
**Number of perceived unmet needs**	None reported (Ref.)		
	**1−4 unmet needs**	−0.23 (− 0.46 to 0.00)	**−0.28 (− 0.52 to − 0.04)**
	**≥5 unmet needs**	**−0.75 (− 1.19 to − 0.31)**	**−0.83 (− 1.28 to − 0.38)**
Size of care network	2−3 providers (Ref.)		
	4−5 providers	0.07 (− 0.19 to 0.32)	0.13 (− 0.14 to 0.39)
	6−10 providers	0.04 (− 0.28 to 0.36)	0.11 (− 0.23 to 0.44)

CI, confidence interval; CSHCN, children with special health care needs; β, regression coefficient

Regression coefficients (β) with 95% confidence intervals (CI) based on pooled estimates from 40 imputed datasets are reported (*n* = 459). Associations were considered statistically significant when the 95% CI excluded zero and are highlighted in bold

**Table 3:** Associations between caregiver-reported *Attention to family impact* and sociodemographic, health- and network-related potential correlates

		2a: Univariable linear regression models	2b: Multivariable linear regression model
		β (95% CI)	β (95% CI)
Sex of caregiver	Female (Ref.)		
	Male	−0.36 (− 0.72 to 0.01)	−0.29 (− 0.66 to 0.08)
Age of caregiver	≤35 years (Ref.)		
	36–40 years	−0.05 (− 0.37 to 0.27)	0.03 (− 0.30 to 0.36)
	41–45 years	−0.30 (− 0.62 to 0.02)	−0.28 (− 0.61 to 0.05)
	≥46 years	−0.27 (− 0.61 to 0.06)	−0.35 (− 0.73 to 0.02)
German as first language	Yes (Ref.)		
	No	−0.13 (− 0.49 to 0.23)	−0.22 (− 0.58 to 0.14)
**Mothers’ highest level of education**	Basic and intermediate (Ref.)		
	**High**	**−0.36 (− 0.62 to − 0.10)**	−0.24 (− 0.52 to 0.05)
**Fathers’ highest level of education**	Basic and intermediate (Ref.)		
	**High**	**−0.39 (− 0.66 to − 0.11)**	−0.26 (− 0.56 to 0.05)
Sex of CSHCN	Female (Ref.)		
	Male	0.08 (− 0.16 to 0.32)	0.01 (− 0.23 to 0.26)
Age of CSHCN	1–5 years (Ref.)		
	6–11 years	−0.02 (− 0.29 to 0.25)	−0.09 (− 0.38 to 0.19)
	≥12 years	0.01 (− 0.34 to 0.35)	0.13 (− 0.26 to 0.52)
**Reason for seeking care**	Physical impairment (Ref.)		
	**Cognitive impairment**	**0.45 (0.13 to 0.76)**	**0.36 (0.03 to 0.69)**
	Physical and cognitive impairment	0.11 (− 0.18 to 0.41)	−0.01 (− 0.31 to 0.30)
	Only diagnostic assessments and therapies	−0.02 (− 0.51 to 0.47)	−0.19 (− 0.70 to 0.31)
Number of perceived unmet needs	None reported (Ref.)		
	1−4 unmet needs	0.03 (− 0.19 to 0.26)	−0.02 (− 0.24 to 0.21)
	≥5 unmet needs	−0.27 (− 0.69 to 0.16)	−0.37 (− 0.79 to 0.05)
**Size of care network**	2−3 providers (Ref.)		
	**4−5 providers**	**0.30 (0.05 to 0.54)**	**0.33 (0.08 to 0.58)**
	**6−10 providers**	**0.34 (0.03 to 0.65)**	**0.38 (0.07 to 0.70)**

CI, confidence interval; CSHCN, children with special health care needs; β, regression coefficient

Regression coefficients (β) with 95% confidence intervals (CI) based on pooled estimates from 40 imputed datasets are reported (*n* = 459). Associations were considered statistically significant when the 95% CI excluded zero and are highlighted in bold

### Sensitivity analyses

Sensitivity analyses indicated robust results. Given negligible clustering by SPC for both outcomes (*Team quality and communication*: ICC = 0.01; *Attention to family impact*: ICC ≈ 0) and the absence of significant SPC fixed effects, we present the linear regression models as the primary analyses. Results were consistent across pandemic periods in Germany (data not shown).

The complete-case analysis yielded results that were largely consistent with those of the primary multiple imputation analysis. Associations of unmet needs with *Team quality and communication*, as well as those of cognitive impairment and larger care network size with *Attention to family impact*, remained statistically significant. Notably, an association between high maternal education and lower *Attention to family impact* was observed only in the complete-case analysis (β=−0.32, 95% CI: −0.63 to − 0.01) and not in the multiple imputation analysis (β=−0.24, 95% CI: −0.52 to 0.05). This difference suggests that the complete-case estimate may have been influenced by selective non-response among caregivers with higher educational attainment. Detailed results are provided in Additional file 3.

## Discussion

In this nationwide multicenter cross-sectional analysis among caregivers of CSHCN with predominantly neurological, developmental, and behavioral conditions in Germany, caregiver-reported EIC was characterized by moderate *Team quality and communication* and low *Attention to family impact*. *Team quality and communication* captures caregivers’ perceptions of provider communication, information sharing across the care team, and family involvement in care decisions. *Attention to family impact* reflects whether providers consider how care decisions affect family burden and daily life beyond the child’s medical needs. The observed pattern suggests that, although caregivers perceived cross-sectoral teams as reasonably well coordinated and communicative, the broader family-level consequences of care decisions remained less well-addressed. Distinct correlates emerged for the two dimensions of integrated care. Unmet needs were the only correlate of *Team quality and communication*, whereas *Attention to family impact* varied by impairment type and care network size. These findings underscore that the two dimensions of caregiver-reported EIC may be shaped by different mechanisms and should be considered separately when developing integrated care interventions for CSHCN.

For *Team quality and communication*, a higher number of unmet needs was the only statistically significant correlate, with a dose-response pattern indicating that families with greater unmet needs perceived lower communication quality. Given the cross-sectional design, the direction of this association remains unclear. Unmet needs may reflect broader system-level fragmentation, including insufficient case management, that also affects communication. Conversely, limited communication within care teams may contribute to unmet needs by reducing families’ access to information about available services and entitlements [16, 42]. Together, these findings suggest that perceived care integration may be closely linked to needs-based service delivery [43, 44]. Systematic screening for unmet needs may therefore help identify families who could benefit from enhanced care coordination. Future studies should examine shared determinants of unmet needs and communication quality, including the role of caregiver and provider health literacy, to inform interventions that simultaneously strengthen care coordination and needs-based service delivery [45, 46].

In contrast, the *Attention to family impact* score was associated with a different set of factors. Caregivers of children with cognitive, compared with physical, impairments reported higher scores, possibly reflecting greater attention to family-level concerns among providers caring for children who require extensive cross-sectoral coordination. Contrary to care coordination literature suggesting that involvement of multiple providers contributes to fragmentation [10], larger care network size was associated with higher *Attention to family impact* scores in this study. This association was not observed for *Team quality and communication*, suggesting that the benefit of larger care networks may lie less in improved coordination than in greater attention to family-level needs. One plausible explanation is that care networks for children with cognitive and developmental conditions more often include educational and social services, thereby exposing families to professionals whose roles explicitly encompass family support [4]. Similarly, larger care networks may include a broader range of non-medical professionals who address family-level concerns. However, this interpretation requires further investigation, as previous research has shown that families of children with greater care complexity often report higher unmet needs rather than greater attention to family impact [47]. Our findings generate the hypothesis that integrated care models intentionally incorporating educational, social, and other non-medical professionals alongside medical providers may improve caregiver-reported EIC beyond approaches focused primarily on coordination within the health care sector. Because provider composition was not directly examined, this hypothesis should be evaluated in prospective studies.

In the complete-case sensitivity analysis, an association between high maternal education and lower *Attention to family impact* emerged but was attenuated in the multiple imputation analysis. This attenuation suggests that the association observed in the complete-case analysis may have been influenced by selective response among more highly educated caregivers, who tend to participate more consistently in health surveys [48]. Previous research has shown that caregivers with higher educational attainment report lower satisfaction with care experiences [49], possibly because they navigate complex care systems more actively, identify shortcomings more readily, and hold higher expectations. Similarly, evidence from the PART-CHILD cohort suggests that caregivers with higher education report more unmet needs, potentially reflecting greater awareness of available services and entitlements rather than objectively poorer access (unpublished data). Although our findings are consistent with this, the association was not robust to multiple imputation and should therefore be interpreted cautiously and confirmed in future studies. From a health services research perspective, the sensitivity findings suggest that higher socioeconomic resources may not necessarily translate into more positive care experiences, and satisfaction-based outcome measures may be influenced by caregiver expectations. If confirmed, clearer communication about care plans and decision-making processes may help align expectations across educational backgrounds.

The modest explained variance of our models (6.4% and 10.3%) suggests that caregiver-reported EIC is substantially influenced by factors beyond the characteristics examined in this study, including provider-level factors (e.g., attitudes toward family engagement) and system-level factors (e.g., reimbursement models that incentivize coordination). Multilevel frameworks for complex care emphasize this interplay of micro-, meso-, and macro-level determinants [50]. The differing patterns of associations for the two composite scores further suggest that communication and collaboration versus attention to family impact within care networks may be influenced by different factors. If confirmed in future studies, this distinction could have implications for the design of integrated care interventions and the selection of outcome measures [7].

The study has several strengths. Sensitivity analyses indicated that the main findings were not driven by influential observations and were generally consistent across alternative model specifications accounting for potential clustering within SPC. The sample demographics largely reflected known population patterns. The higher proportion of male children is consistent with the sex-specific prevalence of developmental and behavioral conditions [47, 51]. Furthermore, the study draws on a large, nationwide multicenter sample and uses a validated instrument specifically designed to capture different dimensions of caregiver-reported EIC.

However, several limitations should be acknowledged. First, the cross-sectional design precludes causal inference, and the observed associations may be bidirectional, particularly those between unmet needs and *Team quality and communication*. Second, missing values on regression covariates were addressed using multiple imputation. Although this approach is considered robust under the missing-at-random assumption and reduces the risk of selection bias associated with complete-case analysis, residual bias due to unobserved differences between responders and non-responders cannot be excluded. The complete-case sensitivity analysis confirmed the direction of the main associations and additionally suggested that the association between *Attention to family impact* and maternal education observed in complete cases may partly reflect selective non-response among more highly educated caregivers. Third, data were exclusively collected in SPC, which primarily provide care for children with neurodevelopmental and behavioral conditions. This population has limited capacity to compensate for fragmented care delivery [6]. Consequently, the findings may not generalize to CSHCN with primarily somatic conditions or those receiving care in child and adolescent psychiatry settings. Future research should include more diverse populations of CSHCN and care settings. Finally, data collection coincided with the early phases of the COVID-19 pandemic, which likely affected service delivery, provider communication, and family burden in ways not fully captured by our pandemic-phase stratification. Although sensitivity analyses indicated stable associations across pandemic periods in Germany [41], residual effects on absolute caregiver-reported EIC levels cannot be ruled out.

Further limitations relate to the measurement of key variables. Sociodemographic characteristics were assessed with limited precision. Age was collected in categories due to data protection regulations, and educational and vocational background was assessed only to a limited extent according to established guidelines to reduce participant burden, limiting the precision of the ISCED classification [52]. The associations between caregiver-reported EIC and maternal and paternal education should therefore be interpreted with caution. Because children’s living arrangements were not assessed, the reported education may not correspond to that of the caregiver primarily responsible for the child’s daily care. Furthermore, a slightly earlier version of the PICS-D was used because the final validated version was published only after data collection (Additional file 1). No information on impairment severity was collected, limiting the interpretation of findings across clinical subgroups [53]. In addition, no standardized CSHCN screener was used (e.g [54]), precluding verification of whether individual children met standardized CSHCN criteria. Although information on care network composition was available, it lacked details on specific provider types. Future research should adopt a more comprehensive care network perspective, including social and educational services and system-level factors, to improve understanding of the determinants of caregiver-reported EIC.

## Conclusions

Among caregivers of CSHCN participating in the PART-CHILD study, respondents reported moderate *Team quality and communication* and substantial gaps in *Attention to family impact*. Unmet needs emerged as a key correlate of lower perceived *Team quality and communication*, whereas *Attention to family impact* was associated with the child’s type of impairment and care network size.

These findings suggest that caregiver-reported EIC may require greater attention to family-level concerns while accounting for heterogeneity in care needs and care networks. Strengthening health literacy at both the individual and organizational level may complement coordination efforts by improving system navigability and information accessibility. The differing patterns of associations observed for the two composite scores indicate that communication and collaboration and attention to family impact may represent distinct dimensions of caregiver-reported EIC that could benefit from tailored approaches in future research and integrated care interventions. Whether policy incentives that promote cross-sectoral collaboration across health care, educational, and social services can facilitate the implementation of integrated care at the system level warrants further study.

## Supplementary Information

Below is the link to the electronic supplementary material.

Supplementary Material 1

Supplementary Material 2

Supplementary Material 3

## Data Availability

The datasets used and/or analyzed during the current study are available from the corresponding author upon reasonable request.

## Abbreviations

CHC-SUN: Child Health Care–Satisfaction, Utilization and Needs
CI: Confidence interval
CSHCN: Children with special health care needs
EIC: Experience of integrated care
ICC: Intraclass correlation coefficient
ISCED: International Standard Classification of Education
MICE: Multiple imputation by chained equations
PICS-D: German Pediatric Integrated Care Survey
SD: Standard deviation
SPC: Social Pediatric Center
VIF: Variance inflation factor
