# Supplementary material for: Caregiver-reported experience of integrated care for children with special health care needs and its sociodemographic, health- and network-related correlates in Germany: Cross-sectional results from the PART-CHILD cohort
Source: BMC Health Serv Res. 2026 Aug 8;26:1077. doi: 10.1186/s12913-026-15276-6 (PMC13455375; doi:10.1186/s12913-026-15276-6)
Supplement: Supplementary file 1 [file 12913_2026_15276_MOESM1_ESM.docx]

# Additional files

## Additional file 1: Overview of collected data

Table S1: Overview of all variables and their operationalization

| **Construct / Variable** | **Description / Item source** | **Type** | **Coding** | **Operationalization** | **BL** | **FU** |
| --- | --- | --- | --- | --- | --- | --- |
| **Dependent variables** | | | | | | |
| *Team quality & communication* | Perceived quality of cooperation within the care network, team communication and involvement in care decisions (PICS-D composite score [24]) | Likert-scale | 1=never; 2=rarely; 3=sometimes; 4=usually; 5=almost always; 6=always | Mean score of 7 items; higher scores = better perception |  | x |
| *Attention to family impact* | Perceived communication on impact of care decisions on the entire family (PICS-D composite score [24]) | Likert-scale | 1=never; 2=rarely; 3=sometimes; 4=usually; 5=almost always; 6=always | Mean score of 4 items; higher scores = better perception |  | x |
| **Independent variables (sociodemographic)** | | | | | | |
| Caregiver characteristics | | | | | | |
| Gender | Self-reported gender of responding caregiver | Binary | 1=male; 2=female |  | x |  |
| Age | Self-reported age of responding caregiver (in years) | Categorical | 1=≤19; 2=20–26; 3=27–31; 4=32–36 years; 5=37–41; 6=42–46; 7=≥47 |  | x |  |
| Highest level of education | Self-reported educational background of mother and father | Ordinal | 1=No school qualification; 2=Secondary school diploma; 3=Secondary school diploma, polytechnic; secondary school diploma, or 10th grade completed; 4=Advanced secondary school certificate, technical college entrance qualification, or high school diploma; 5=Cannot specify | categorized by the ISCED levels education [25]: 1=basic; 2=intermediate; 3=high | x |  |
| Highest level of vocational training | Self-reported vocational background of mother and father | Ordinal | 1=No vocational qualification (and not currently in training); 2=Currently in vocational training (e.g., student, apprentice, pre-vocational year, intern); 3=Completed vocational training or apprenticeship; 4=Technical school (e.g., master craftsman, technician school, vocational or professional academy); 5=University of applied sciences, engineering school; 6=Bachelor’s degree, university of applied sciences diploma; 7=Master’s degree, Magister, university diploma, or doctoral degree; 8=Cannot specify | categorized by the ISCED levels [25]: 1=basic; 2=intermediate; 3=high | x |  |
| German as first language | Self-reported short assessment of first language | Binary | 0 = no; 1 = yes |  | x |  |
| Child characteristics | | | | | | |
| Gender | Caregiver-reported gender of child | Binary | 1=male; 2=female |  | x |  |
| Age | Caregiver-reported age of child (in years) | Categorical | 1=1–3; 2=4–5; 3 =6–7; 4=8–11; 5=>11 |  | x |  |
| **Health- and network-related correlates** | | | | | | |
| Type of impairment | Caregiver-reported primary reason for seeking care in SPC | Free text |  | categorized by 1=Physical impairment; 2=Cognitive impairment: 3=Physical & cognitive impairment; 4=Only diagnostic assessments & therapies |  | x |
| Unmet needs | Child Health Care-Satisfaction, Utilization and Needs (CHC-SUN) [28] | Discrete | 1=Physiotherapy; 2=Occupational therapy; 3=Speech therapy; 4=Supply with medical equipment; 5=Supply with physical aids; 6=Social worker services; 7=Psychological counseling; 8=Health education; 9=Coordination of services; 10=Self-help groups; 11=Rehabilitation services; 12=Short-term respite care; 13=Nursing services at home; 14=Telephone counseling; 15=Health services at school/daycare center; 16=Dietary counseling | Count of reported unmet needs |  | x |
| Size of care network | Number of providers involved in child’s care network | Discrete | 1=pediatrician; 2=general practitioner; 3=medical specialists; 4=therapists (e.g. physiotherapist); 5=medical support at home; 6=medical support in educational setting; 7=behavioral medicine practitioners; 8=complementary and alternative medicine practitioners; 9=members of the social services team; 10=social services and youth welfare office staff; 11=educators in kindergarten; 12=teacher at school; 13=Integration assistance in kindergarten or school; 14=free text field | Count of involved providers |  | x |
| **Data collection** | Data collection during COVID-19 pandemic | Categorical | 1=period 0+1 (spring 2020); 2=period 2+3 (summer–autumn 2020) | According to [32] |  | x |

BL, baseline survey; FU, first follow-up survey

Table S2: PICS-D items of dimension Team quality & communication combining original constructs ‘communication’ (items 22, 25, 32) and ‘team functioning’ (items 23, 26, 28, 29)

| **Item#** | **German Version of PICS (PICS-D) with modification in the PART-CHILD cohort:**  **Beziehen Sie sich bitte auf die vergangenen 12 Monate.** | **Original PICS in English:**  **In the past 12 months,** |
| --- | --- | --- |
| 22 | Wie oft haben die Mitglieder des Versorgungsnetzes Ihnen Dinge so erklärt, dass Sie sie verstehen konnten?  z.B. medizinische Begriffe, Therapiedurchführung, Anwendung von Hilfsmitteln | … how often did your child’s care team members explain things in a way that you could understand? |
| 23 | Wie oft hatten Sie das Gefühl, dass Behandlungsempfehlungen zwischen den Mitgliedern des Versorgungsnetzes weitergegeben wurden? | … how often did you feel that your child’s care team members knew about the advice you got from your child’s other care team members? |
| 25 | Wie oft hatten Sie das Gefühl, dass Sie von den Mitgliedern des Versorgungsnetzes gehört wurden, wenn Sie etwas ~~über die~~ zur Gesundheit Ihres Kindes zu sagen hatten? | … how often did you feel that your child’s care team members listened carefully to what you had to say about your child’s health and care? |
| 26 | Wie oft ~~wurde mit Ihnen besprochen~~ hat ein Mitglied des Versorgungsnetzes Ihnen erklärt, wer für die verschiedenen Bereiche der Versorgung und Betreuung Ihres Kindes verantwortlich ist? | … how often has someone on your child’s care team explained to you who was responsible for different parts of your child’s care? |
| 28 | Wie oft hatten Sie das Gefühl, dass die Mitglieder des Versorgungsnetzes ihre ~~Aufgaben~~ Verantwortung in der Versorgung und Betreuung Ihres Kindes ~~erfüllten~~ voll und ganz wahrgenommen haben? | … how often did your feel that your child’s care team members followed through with their responsibilities related to your child’s care? |
| 29 | Wie oft hatten Sie das Gefühl, dass die Mitglieder des Versorgungsnetzes ~~bei der Betreuung Ihres Kindes seine Gesamtsituation berücksichtigt haben~~ ihr Kind ganzheitlich betrachtet haben, d.h. alle Bedürfnisse Ihres Kindes? | … how often have you felt that your child’s care team members thought about the “big picture” when caring for your child, meaning dealing with all of your child’s needs? |
| 32 | Wie oft haben die Mitglieder des Versorgungsnetzes Sie ~~als vollwertige/n Partner/in~~ in der Versorgung und Betreuung Ihres Kindes als Partner auf Augenhöhe behandelt? | … how often have your child’s care team members treated you as a full partner in the care of your child? |

The items are Likert-scaled and range from 1 to 6: 1=‘never’; 2=‘rarely’; 3=‘sometimes’; 4=‘usually’; 5=‘almost always’; 6=‘always’. German Version: 1=‘nie‘; 2=‘selten‘; 3=‘manchmal‘; 4=‘~~meistens~~ normalerweise‘; 5=‘fast immer’; 6=‘immer.

Table S3: PICS-D items of dimension Attention to family impact

| **Item#** | **German Version of PICS (PICS-D) with modification in the PART-CHILD cohort:**  **Beziehen Sie sich bitte auf die vergangenen 12 Monate.** | **Original PICS in English:**  **In the past 12 months,** |
| --- | --- | --- |
| 33 | Wie oft haben die Mitglieder des Versorgungsnetzes mit Ihnen darüber gesprochen, wie sich Entscheidungen ~~über die~~ zur Versorgung Ihres Kindes auf Ihre ganze Familie auswirken ~~werden~~? | … how often have your child’s care team members talked with you about how health care decisions for your child will affect your whole family? |
| 34 | Wie oft haben die Mitglieder des Versorgungsnetzes mit Ihnen über Belastungen gesprochen, die sich für Sie durch die Erkrankung und den Betreuungsbedarf Ihres Kindes ergeben ~~können~~? | … how often have your child’s care team members talked to you about things in your life that cause you stress because of your child’s health or care needs? |
| 35 | Wie oft haben die Mitglieder des Versorgungsnetzes Situationen angesprochen, die es Ihnen schwer machen ~~können~~, sich um die Gesundheit Ihres Kindes zu kümmern?  (z.B. Arbeit, eigene Krankheit etc.)~~?~~ | … how often have your child’s care team members talked to you about things that make it hard for you to take care of your child’s health? |
| 37 | Wie oft haben ~~Ihnen~~ die Mitglieder des Versorgungsnetzes Ihnen die Möglichkeit ~~an~~geboten, mit anderen betroffenen Familien in Kontakt zu treten? | … how often have your child’s care team members offered you opportunities to connect with other families who they thought might be of help to you? |

The items are Likert-scaled and range from 1 to 6: 1=‘never’; 2=‘rarely’; 3=‘sometimes’; 4=‘usually’; 5=‘almost always’; 6=‘always’. German Version: 1=‘nie‘; 2=‘selten‘; 3=‘manchmal‘; 4=‘~~meistens~~ normalerweise‘; 5=‘fast immer’; 6=‘immer.

Table S4: Additional PICS-D items: not included in scoring

| **Item#** | **German Version of PICS (PICS-D) with modification in the PART-CHILD cohort:**  **Beziehen Sie sich bitte auf die vergangenen 12 Monate.** | **Original PICS in English:**  **In the past 12 months, …** |
| --- | --- | --- |
| 30 | Haben die Mitglieder des Versorgungsnetzes Ihres Kindes kurzfristige Behandlung- oder Betreuungsziele ~~für Ihr Kind gesetzt~~ festgelegt, ~~d.h. Ziele~~ die für bis zu 6 Monaten ~~in der Zukunft~~ gelten? ^a^  z.B. Medikamente, Ziele der körperlichen und sprachlichen Entwicklung, gemeinsames Spielen mit anderen Kindern, Teilnahme an gemeinsamen Aktivitäten im Kindergarten | have your child’s care team members created short-term care goals, meaning goals up to 6 months in the future? |
| 31 | Haben die Mitglieder des Versorgungsnetzes Ihres Kindes kurzfristige Behandlung- oder Betreuungsziele ~~für Ihr Kind gesetzt~~ festgelegt, ~~d.h. Ziele~~ die für 6 Monate oder länger ~~in der Zukunft~~ gelten? ^a^  z.B. Medikamente, Ziele der körperlichen und sprachlichen Entwicklung, gemeinsames Spielen mit anderen Kindern, Teilnahme an gemeinsamen Aktivitäten im Kindergarten | have your child’s care team members created long-term care goals, meaning goals 6 months or longer into the future? |
| 13 | Hatten alle ~~Behandelnden~~ Mitglieder des Versorgungsnetzes Zugang zu den für sie wichtigen medizinischen Unterlagen (z.B. Arztbriefe)? | did all of your child’s medical providers have access to the same medical records? |
| 17 | Wie oft hatten Sie in den letzten 12 Monaten aufgrund von Wartezeiten oder anderen Problemen Schwierigkeiten ~~bzgl.~~, für Ihr Kind einen Termin bei einem medizinische~~r~~n oder sozialer~~r~~nDienste ~~(z.B. Therapien, Integrationshilfe etc.), weil es Wartelisten oder andere Probleme bei der Terminvergabe gab~~ zu bekommen? ^b^  z.B. Arzt, Therapeut, Pflegedienst, Sozialstation, Integrationshilfe | how often did you have difficulties or delays getting medical or social services for your child because there were waiting lists, backlogs, or other problems getting appointments? |
| 18 | Manchmal wissen Familien in Ihrer Situation gar nicht,  wer für die Belange ihres Kindes der richtige Ansprechpartner ist.  Wie oft hatten Sie Schwierigkeiten ~~bzgl. medizinischer oder sozialer Dienste (z.B. Therapien, Integrationshilfe etc.)~~, ~~weil Sie nicht wussten, wer der~~ die richtige Anlaufstelle oder den richtigen Ansprechpartner ~~ist~~ bei einem medizinischen oder sozialen Dienst zu finden? ^b^  z.B. Arzt, Therapeut, Pflegedienst, Sozialstation, Integrationshilfe | how often did you have difficulties or delays getting medical or social services for your child because you had trouble getting the information you needed? |
| 36 | Wie oft haben die Mitglieder des Versorgungsnetzes angeboten, ~~mit Ihnen~~ auch auf andere Weise als durch einen persönlichen Besuch mit Ihnen zu kommunizieren ~~(z.B. Telefon, E-Mail oder Skype)~~, wenn keine körperliche Untersuchung ~~erforderlich war~~ notwendig war?  z.B. Telefon, E-Mail, Skype | how often have your child’s care team members offered to communicate with you in ways other than an in-person visit, such as phone, email, skype or telehealth, if no physical examination was necessary? |
| 24 | ~~Manchmal haben Eltern Bedenken zur Gesundheit und Versorgung Ihres Kindes.~~ Wie oft ist ~~es Ihnen leicht gefallen, den~~ konnten Sie sich gegenüber Mitgliedern des Versorgungsnetzes ~~Ihre Bedenken mitzuteilen~~ öffnen und ihnen mitteilen, dass Sie Sorgen wegen der Gesundheit oder der Betreuung Ihres Kindes hatten? ^b^ | how often did you feel comfortable letting your child’s care team members know that you had any concerns about your child’s health or care? |
| 27 | Wie oft hatten Sie das Gefühl, dass die Mitglieder des Versorgungsnetzes über alle bisherigen Tests und Untersuchungen ~~Ihres Kindes informiert waren~~ Bescheid wussten, um unnötige weitere Untersuchungen zu vermeiden? ^b^ | how often did you feel that your child’s care team members were aware of all tests and evaluations your child has had recently in order to avoid unnecessary testing? |

^a^ Two answer modalities: ‘yes’ or ‘no’.

^b^ The items are Likert-scaled and range from 1 to 6: 1=‘never’; 2=‘rarely’; 3=‘sometimes’; 4=‘usually’; 5=‘almost always’; 6=‘always’. German Version: 1=‘nie‘; 2=‘selten‘; 3=‘manchmal‘; 4=‘~~meistens~~ normalerweise‘; 5=‘fast immer’; 6=‘immer.
