# Supplementary material for: Caregiver-reported experience of integrated care for children with special health care needs and its sociodemographic, health- and network-related correlates in Germany: Cross-sectional results from the PART-CHILD cohort
Source: BMC Health Serv Res. 2026 Aug 8;26:1077. doi: 10.1186/s12913-026-15276-6 (PMC13455375; doi:10.1186/s12913-026-15276-6)
Supplement: Supplementary file 2 [file 12913_2026_15276_MOESM2_ESM.docx]

**Additional file 2**

**Overview of variables included in the multiple imputation model**

The multiple imputation model was estimated using the mice package in R (chained equations, m = 40 imputations, predictive mean matching for continuous variables, logistic or polytomous regression for binary and categorical variables). The following variables were included as outcomes, regression covariates, and auxiliary variables. Auxiliary variables were not part of the regression models but were expected to correlate with the missingness mechanism and with the variables to be imputed.

| **Variable** | **Description** | **Format** |
| --- | --- | --- |
| **Outcomes** | | |
| pics_team_comp_score | Team quality & communication composite score (PICS-D) | numeric |
| pics_fam_comp_score | Attention to family impact composite score (PICS-D) | numeric |
| **Regression covariates** | | |
| T1_sex_par | Sex of caregiver | factor (2 levels) |
| T1_age_par_reg | Age of caregiver (categorical) | factor |
| T1_ger_l | German as first language | factor (2 levels) |
| T1_ISCED_m_reg | Mother's highest level of education (ISCED) | factor |
| T1_ISCED_f_reg | Father's highest level of education (ISCED) | factor |
| T1_sex_child | Sex of CSHCN | factor (2 levels) |
| T1_age_child_reg | Age of CSHCN (categorical) | factor |
| T2_ind_cat_lbl | Reason for seeking care | factor |
| T1_chc19_unmet_grp2 | Number of perceived unmet needs (categorical) | factor |
| T2_pics0_Anzahl_reg | Size of care network (categorical) | factor |
| **Auxiliary variables** | | |
| **Design and respondent characteristics** | | |
| spz_id | Social pediatric center (SPC) identifier | factor |
| T2_spz_y | Duration of care received at the SPC (years since first contact) | numeric |
| T2_FB_eingang_COVID | Pandemic phase in Germany at questionnaire completion (1 = first COVID-19 wave, 2 = second COVID-19 wave) | factor |
| T1T2_fillout_all | Respondent type (mother, father, other caregivers) | factor |
| **Non-validated PICS-D items (MCN = members of the care network)** | | |
| T2_pics1 | PICS-D #17: Caregiver had difficulty getting appointments * | numeric |
| T2_pics2 | PICS-D #18: Caregiver had difficulty finding the right information * | numeric |
| T2_pics3 | PICS-D #36: MCN offered alternative communication modes (e.g., email) * | numeric |
| T2_pics4 | PICS-D #27: MCN was aware of past tests / avoiding duplication * | numeric |
| T2_pics5_4 | PICS-D #30: MCN created short-term care goals (yes / no) | factor (2 levels) |
| T2_pics5_7 | PICS-D #31: MCN created long-term care goals (yes / no) | factor (2 levels) |
| T2_pics6 | PICS-D #13: MCN had access to same medical records (yes / no) | factor (2 levels) |
| T2_pics7_3 | PICS-D #24: Caregivers are able to share concerns about child's care openly * | numeric |
| **Caregiver-reported satisfaction with care (CHC-SUN items)** | | |
| chc3 | Satisfaction with primary pediatric care ** | numeric |
| chc7 | Satisfaction with SPC-based care ** | numeric |
| chc14 | Satisfaction with emergency outpatient care ** | numeric |

CHC-SUN, Child Health Care–Satisfaction, Utilization and Needs; CSHCN, children with special health care needs; ISCED, International Standard Classification of Education; MCN, members of the care network; PICS-D, German Pediatric Integrated Care Survey; SPC, Social Pediatric Center.

* Items were rated on a six-point Likert scale: never, rarely, sometimes, usually, almost always, always.

** Satisfaction items were rated on a five-point Likert scale: not satisfied, partly satisfied, satisfied, very satisfied, extremely satisfied.
