# Supplementary material for: Caregiver-reported experience of integrated care for children with special health care needs and its sociodemographic, health- and network-related correlates in Germany: Cross-sectional results from the PART-CHILD cohort
Source: BMC Health Serv Res. 2026 Aug 8;26:1077. doi: 10.1186/s12913-026-15276-6 (PMC13455375; doi:10.1186/s12913-026-15276-6)
Supplement: Supplementary file 3 [file 12913_2026_15276_MOESM3_ESM.docx]

**Additional file 3**

**Comparison of multiple imputation and complete-case analyses for the two multivariable linear regression models**

Multiple imputation was performed using chained equations (mice package in R) on the analytic sample of caregivers with care networks of two or more providers (*n* = 459). Forty imputed datasets were generated, and parameter estimates were pooled according to Rubin's rules. Complete-case analysis was restricted to caregivers with no missing values on any regression variable (*n* = 335).

**Model 1: Team quality & communication**

| **Variable** | **Category** | **Multiple imputation**  **(*n* = 459, m = 40)**  **β (95% CI)** | **Complete-case**  **(*n* = 335)**  **β (95% CI)** |
| --- | --- | --- | --- |
| Sex of caregiver | Female (Ref.) |  |  |
|  | Male | −0.21 (−0.63 to 0.21) | 0.02 (−0.46 to 0.50) |
| Age of caregiver | ≤35 years (Ref.) |  |  |
|  | 36–40 years | 0.03 (−0.31 to 0.38) | 0.09 (−0.26 to 0.44) |
|  | 41–45 years | −0.03 (−0.38 to 0.32) | 0.04 (−0.32 to 0.41) |
|  | ≥46 years | −0.27 (−0.66 to 0.13) | −0.16 (−0.57 to 0.26) |
| German as first language | Yes (Ref.) |  |  |
|  | No | −0.13 (−0.53 to 0.27) | −0.17 (−0.56 to 0.21) |
| Mother's education | Basic & intermediate (Ref.) |  |  |
|  | High | −0.08 (−0.39 to 0.23) | −0.29 (−0.60 to 0.03) |
| Father's education | Basic & intermediate (Ref.) |  |  |
|  | High | −0.20 (−0.52 to 0.11) | −0.09 (−0.41 to 0.23) |
| Sex of CSHCN | Female (Ref.) |  |  |
|  | Male | −0.06 (−0.33 to 0.20) | −0.06 (−0.34 to 0.23) |
| Age of CSHCN | 1–5 years (Ref.) |  |  |
|  | 6–11 years | −0.03 (−0.33 to 0.27) | −0.01 (−0.31 to 0.30) |
|  | ≥12 years | 0.13 (−0.28 to 0.54) | 0.26 (−0.18 to 0.70) |
| Reason for seeking care | Physical impairment (Ref.) |  |  |
|  | Cognitive impairment | −0.07 (−0.42 to 0.28) | −0.01 (−0.38 to 0.35) |
|  | Physical and cognitive impairment | −0.24 (−0.56 to 0.08) | −0.32 (−0.66 to 0.02) |
|  | Only diagnostic assessments and therapies | −0.22 (−0.75 to 0.32) | −0.32 (−0.89 to 0.25) |
| Number of perceived unmet needs | None reported (Ref.) |  |  |
|  | **1–4 unmet needs** | **−0.28 (−0.52 to −0.04)** | **−0.32 (−0.58 to −0.06)** |
|  | **≥5 unmet needs** | **−0.83 (−1.28 to −0.38)** | **−0.86 (−1.31 to −0.41)** |
| Size of care network | 2–3 providers (Ref.) |  |  |
|  | 4–5 providers | 0.13 (−0.14 to 0.39) | 0.08 (−0.20 to 0.37) |
|  | 6–10 providers | 0.11 (−0.23 to 0.44) | 0.16 (−0.20 to 0.52) |

CI, confidence interval; CSHCN, children with special health care needs; β, regression coefficient.

Bold entries indicate associations for which the 95% CI excluded zero in both the complete-case and multiple imputation analyses.

**Model 2: Attention to family impact**

| **Variable** | **Category** | **Multiple imputation**  **(*n* = 459, m = 40)**  **β (95% CI)** | **Complete-case**  **(*n* = 335)**  **β (95% CI)** |
| --- | --- | --- | --- |
| Sex of caregiver | Female (Ref.) |  |  |
|  | Male | −0.29 (−0.66 to 0.08) | −0.19 (−0.66 to 0.28) |
| Age of caregiver | ≤35 years (Ref.) |  |  |
|  | 36–40 years | 0.03 (−0.30 to 0.36) | 0.03 (−0.32 to 0.37) |
|  | 41–45 years | −0.28 (−0.61 to 0.05) | −0.24 (−0.59 to 0.12) |
|  | ≥46 years | −0.35 (−0.73 to 0.02) | −0.28 (−0.69 to 0.13) |
| German as first language | Yes (Ref.) |  |  |
|  | No | −0.22 (−0.58 to 0.14) | −0.27 (−0.64 to 0.11) |
| Mother's education | Basic & intermediate (Ref.) |  |  |
|  | High ^1^ | −0.24 (−0.52 to 0.05) | −0.32 (−0.63 to −0.01) |
| Father's education | Basic & intermediate (Ref.) |  |  |
|  | High | −0.26 (−0.56 to 0.05) | −0.19 (−0.50 to 0.13) |
| Sex of CSHCN | Female (Ref.) |  |  |
|  | Male | 0.01 (−0.23 to 0.26) | −0.02 (−0.30 to 0.26) |
| Age of CSHCN | 1–5 years (Ref.) |  |  |
|  | 6–11 years | −0.09 (−0.38 to 0.19) | −0.01 (−0.31 to 0.28) |
|  | ≥12 years | 0.13 (−0.26 to 0.52) | 0.19 (−0.24 to 0.62) |
| Reason for seeking care | Physical impairment (Ref.) |  |  |
|  | **Cognitive impairment** | **0.36 (0.03 to 0.69)** | **0.50 (0.14 to 0.86)** |
|  | Physical and cognitive impairment | −0.01 (−0.31 to 0.30) | −0.09 (−0.43 to 0.24) |
|  | Only diagnostic assessments and therapies | −0.19 (−0.70 to 0.31) | −0.29 (−0.85 to 0.27) |
| Number of perceived unmet needs | None reported (Ref.) |  |  |
|  | 1–4 unmet needs | −0.02 (−0.24 to 0.21) | −0.07 (−0.33 to 0.19) |
|  | ≥5 unmet needs | −0.37 (−0.79 to 0.05) | −0.34 (−0.78 to 0.10) |
| Size of care network | 2–3 providers (Ref.) |  |  |
|  | **4–5 providers** | **0.33 (0.08 to 0.58)** | **0.41 (0.13 to 0.69)** |
|  | **6–10 providers** | **0.38 (0.07 to 0.70)** | **0.47 (0.12 to 0.83)** |

CI, confidence interval; CSHCN, children with special health care needs; β, regression coefficient.

Bold entries indicate associations for which the 95% CI excluded zero in both the complete-case and multiple imputation analyses.

^1^ The association of high maternal education with lower *Attention to family impact* was statistically significant in the complete-case analysis but attenuated and no longer statistically significant under multiple imputation, suggesting sensitivity of this finding to missing data.
